# Exploring Associations Between Infant Attachment, Maternal Sensitivity, and Attention to Maternal Emotion Expressions

**DOI:** 10.1111/desc.70057

**Published:** 2025-07-31

**Authors:** Mikko J. Peltola, Szilvia Biro, Rens Huffmeijer, Hanneli Sinisalo, Marian J. Bakermans‐Kranenburg, Marinus H. van IJzendoorn

**Affiliations:** ^1^ Human Information Processing Laboratory, Psychology, Faculty of Social Sciences Tampere University Tampere Finland; ^2^ Institute of Education and Child Studies Leiden University Leiden the Netherlands; ^3^ William James Center for Research ISPA – University Institute of Psychological, Social and Life Sciences Lisbon Portugal; ^4^ Research Department of Clinical, Education and Health Psychology, Faculty of Brain Sciences University College London London UK; ^5^ Facultad de Psicología y Humanidades Universidad San Sebastián Sede Valdivia Chile

**Keywords:** attachment, attention, emotion, event‐related potentials, face processing, infants

## Abstract

Recent studies have indicated that patterns of infant‐caregiver attachment are associated with differences in infants’ processing of social signals of emotion, such as facial expressions. In the current longitudinal study we extended this line of research to social signals of actual attachment figures by investigating whether 7‐month‐old infants’ neural and attentional responses to their mother's angry and happy facial expressions are associated with infants’ attachment security to the mother at 12 months of age. At 7 months (*n* = 88), we measured event‐related potentials (ERP) to the mother's angry and happy faces and attention dwell times to the mother's and a stranger's angry and happy faces, and maternal sensitivity was assessed during free play. At 12 months (*n* = 69), infant attachment was assessed with the Strange Situation procedure. In infants who were later classified as having secure attachment, the Nc ERP component indexing attention allocation was larger to maternal happy than angry expressions, whereas no difference in Nc amplitudes to maternal facial expressions was observed in infants with later insecure attachment. We speculate that this may indicate greater approach motivation triggered by the reward value of happy faces in securely attached infants through repeated experiences of positive interaction with the caregiver.

**Summary:**

- Studies have indicated that different patterns of infant‐caregiver attachment are associated with infants’ processing of emotion expressions.
- We investigated whether infants’ attachment security is related to attentional and neural responses to their mother's facial expressions.
- Infants with secure attachment displayed larger attention‐related brain responses to their mother's happy than angry faces, no difference was found in insecurely attached infants.
- This may indicate greater reward value associated with happy faces in securely attached infants through repeated experiences of positive interaction with the caregiver.

## 1 Introduction

A key proposition of attachment theory is that through the internal working models shaped by early close relationships, individuals differ in the way they perceive and evaluate social and emotional information (Dykas and Cassidy 2011; Long et al. 2020; Zimmermann and Iwanski 2015). In adults, insecure attachment has been associated with quicker orienting to images depicting social interactions and emotional faces (Maier et al. 2005) but also with a greater tendency to orient attention away from emotionally negative faces (Dewitte and De Houwer 2008). At school age, insecure attachment (Steele et al. 2008) and attachment disorganization (Forslund et al. 2017) have been associated with lower accuracy in identifying facial expressions, and disorganized attachment also with diminished attention to emotional faces (Forslund et al. 2020). Kammermeier et al. (2020) found that looking times to fearful faces in a free‐viewing task were shorter in 5‐year‐old children with insecure attachment. Together these findings highlight attachment‐related individual differences in processing social information across the lifespan and also provide some evidence for Bowlby's (1973; see also Dykas and Cassidy 2011) notion of *defensive exclusion* in insecure attachment, that is, a tendency to suppress attention to stimuli signaling potential threat or distress as a means to regulate emotional arousal.

Studies have also begun to investigate whether attachment‐related differences in emotional information processing manifest already in infancy, during development of infant‐caregiver attachment patterns. In the present study, we extend this line of research by studying whether infants' neural and attentional responses to maternal facial emotion signals are associated with later attachment patterns. When infants have been presented with animated scenarios depicting responsive and unresponsive caregiving interactions between caregiver and child characters, looking time results indicate that 12‐ to 16‐month‐old securely attached infants expect a comforting response following separation (Johnson et al. 2007), and 12‐month‐old securely attached infants focus attention more to the caregiver character than insecurely attached infants (Biro et al. 2015).

Another line of research has investigated whether infants’ attachment is associated with processing facial emotion signals. Using an eye‐tracking task to measure attention disengagement from fearful, happy, and neutral faces, and non‐face stimuli, Peltola et al. (2015) found that 7‐month‐old infants later classified as insecurely attached showed a reduced attention bias to fearful faces, while securely attached infants displayed a robust and an age‐typical (Leppänen et al. 2018; Peltola et al. 2008, Peltola et al. 2013, 2011) attention bias to fearful faces. Particularly in infants later classified as disorganized there was a lack of attention bias to fearful faces (Peltola et al. 2015). Examining infants’ neural responses to facial expressions using electroencephalography (EEG), Peltola et al. (2020) observed that in 7‐month‐old infants later classified as securely attached, cortical event‐related potential (ERP) responses discriminated between fearful and non‐fearful faces, but similar cortical differentiation of fearful from non‐fearful faces was absent in insecurely attached infants. These initial infant data indicate that attachment‐related differences in processing social emotion signals emerge early, preceding the age at which the standard attachment assessment with the Strange Situation procedure (Ainsworth et al. 1978) can be performed (i.e., 10–12 months).

Importantly, as the previous studies demonstrating attachment‐related differences in children's responses to facial emotions (e.g., Forslund et al. 2020; Kammermeier et al. 2020; Peltola et al. 2015) all used face stimuli that were unfamiliar to the children, they do not inform whether similar processes are active within actual attachment relationships. Therefore, to better understand how attachment formation, early interaction and social information processing are intertwined, it is important to investigate whether infants’ responses to emotional signals of attachment figures (e.g., caregiver facial expressions) are related to different patterns of attachment. Especially before language onset, face‐to‐face communication is a key channel for transmitting signals of affiliation, comfort and rejection in caregiver‐infant relationships. Research on infants’ responses to caregiver signals using microanalysis of infant behaviors during face‐to‐face interaction with the mother indicates that insecure infant attachment is predicted by reduced attention towards the mother's face during the first year (Beebe et al. 2010; Koulomzin et al. 2002). No studies, however, have directly investigated infants’ responses to maternal emotion expressions in relation to attachment development. Studying school‐aged children's brain responses to mother and stranger angry and happy faces, Kungl et al. (2023) found that increased levels of attachment deactivation (i.e., avoidance) were associated with an absence of differences in attention‐related ERP responses to maternal happy versus angry expressions, while low deactivation (indicating greater attachment security) was related to larger attention‐related ERP responses to maternal happy versus angry faces. This was considered to reflect reduced sensitivity to maternal reward signals in insecurely attached children (Kungl et al. 2023). Similarly, Vandevivere et al. (2014) found that securely attached school‐aged children looked longer at their mother's face than avoidantly attached children, although no differences in looking at different maternal facial expressions were found.

In the present study, we investigated whether 7‐month‐old infants’ neural and attentional responses to their mother's angry and happy facial expressions are associated with infants’ attachment security to the mother at 12 months of age. Previous studies investigating attachment‐related differences in children's emotion processing have used both fearful (e.g., Peltola et al. 2015) and angry faces (e.g., Kungl et al. 2023) as negative emotion stimuli. To begin studying infants’ processing of maternal emotion expressions, we selected angry faces as the negative emotion stimulus in this study because we considered angry expressions to reflect a direct and unambiguous signal of negative emotion and potential rejection by the attachment figure.

To investigate neural responses, ERP responses reflecting perceptual and attentional processing of maternal facial expressions were measured. The key ERP components associated with face processing in infancy are the occipitotemporal N290 and P400 components, which relate to perceptual detection and discrimination of faces, and likely precede the later‐developing face‐sensitive N170 component, as well as the frontocentral Nc (Negative central) component, which reflects attention allocation (Conte et al. 2020; Yrttiaho et al. 2022). Particularly, the Nc is a useful marker of rapid attention allocation to emotionally salient stimuli. The Nc typically occurs as a sustained negative waveform beginning at around 300 ms, and it has been found to be larger (i.e., more negative) in response to fearful faces (Jessen 2020; Jessen and Grossmann 2015; Leppänen et al. 2007; Peltola et al. 2009; Taylor‐Colls and Fearon 2015) and angry faces (Kobiella et al. 2008; Xie et al. 2019) than happy or neutral faces in infants from 5 to 7 months of age. The Nc component has also been found to be sensitive to face familiarity, with studies pointing to greater Nc responses to maternal faces in infants younger than 12 months (Burden et al. 2007; de Haan and Nelson 1997), followed by a shift to greater Nc responses to unfamiliar faces after 12 months of age (Carver et al. 2003; Luyster et al. 2011).

Studies reporting the N290 and P400 components have also observed modulation of the amplitudes by emotional faces, although the pattern of differences has been mixed. Some studies (e.g., van den Boomen et al. 2019; Xie et al. 2019) have observed more negative amplitudes to fearful than angry or happy faces during the N290 time window (∼200–350 ms), while others have found a reversed pattern (Jessen and Grossmann 2015; Kobiella et al. 2008). Similarly for the P400 component (∼300–500 ms), some studies have observed larger amplitudes to fearful versus happy or angry faces (Jessen and Grossmann 2015; Kobiella et al. 2008; Leppänen et al. 2007), while Xie et al. (2019) observed greater P400 amplitudes to angry than fearful and happy faces. Face familiarity effects on infants’ N290 and P400 responses have received little attention. While no familiarity effects on either component were found by Luyster et al. (2011), Rigato et al. (2025) found that the mother's face triggered larger N290 amplitudes at 4 months and larger P400 amplitudes at 6 months, but the effects were absent at 9 months, resembling the commonly observed absence of face familiarity effects on the N170 ERP component in adults (Bentin and Deouell 2000).

In addition to ERPs, we measured infants’ attention disengagement from both mother and stranger emotional faces with the Overlap task, which is an eye‐movement based task for measuring attention dwell times to centrally presented face stimuli that compete for attention with peripheral distractor stimuli. As described above, attention dwell times to fearful faces measured with the Overlap task have been associated with attachment security (Peltola et al. 2015). We investigated whether a similar association is found between attachment and attention to angry faces, which differ from fearful faces by being unambiguous and potentially threatening signals of negative emotion (Marsh et al. 2005). Another key motivation to include the Overlap task was that in the ERP task we only presented the mother's face stimuli and, therefore, by presenting emotional faces of both the mother and a visually matched stranger we sought to understand whether attachment‐related attention patterns to the emotion stimuli differ based on face familiarity.

As a measure of early caregiving environment, maternal sensitivity during free play interaction with the infant was assessed and taken into account in the analyses. Given the established association between maternal sensitivity and infant attachment security (De Wolff and van IJzendoorn 1997; Verhage et al. 2016), and findings linking responses to facial emotions with infants’ attachment security (Peltola et al. 2015, 2020), it could be expected that infants’ responses to facial emotion signals are shaped by interaction experiences with the caregiver. Currently, there is very little research relating variations in parental sensitivity to infants’ emotion processing, but limited evidence suggests that higher maternal sensitivity may be associated with greater attentiveness to positive emotions in infancy. Taylor‐Colls and Fearon (2015) found that maternal sensitivity was related to larger Nc responses to happy faces, and Stern et al. (2024) observed greater prefrontal fNIRS activation to happy faces in infants with more sensitive mothers. Thus, sensitive interactions fostering secure attachment might particularly relate to infants’ responses to the caregiver's positive emotion signals.

In summary, we investigated whether infants’ neural and attentional responses to maternal angry and happy faces are associated with infants’ attachment security, while controlling for maternal sensitivity. Infants’ neural and attentional responses were measured at 7 months and attachment was assessed at 12 months of age. As the majority of previous research on infants’ attachment‐related emotion processing and ERP responses to faces has centered on 7‐month‐old infants, this age was an optimal time point to study whether later attachment is predicted by emotion processing during the first year. Based on previous research (Kungl et al. 2023; Peltola et al. 2015, 2020), we expected insecure attachment and lower levels of maternal sensitivity to be associated with less pronounced differentiation of maternal emotion signals, reflected in ERP amplitudes and attention dwell times. Regarding how attention dwell times to maternal versus stranger faces may differ in the Overlap task, we did not have specific directional hypotheses. In the main analyses we compared securely attached infants with infants having any type of insecure attachment (avoidant, resistant, disorganized), in line with previous studies (Peltola et al. 2015, 2020). In further exploratory analyses, we compared infants with organized (i.e., secure, avoidant, resistant) versus disorganized patterns of attachment. Finally, in correlational analyses we explored whether the ERP amplitudes and attention dwell times were associated with continuous indices of attachment security (i.e., security score, Van IJzendoorn and Kroonenberg 1990) and organization (i.e., disorganization score).

## 2 Methods

### 2.1 Participants

The infant participants were part of a longitudinal study involving laboratory visits at 7, 10, and 12 months of age (for the 10‐month assessment, see Biro et al. 2021). At 7 months, 88 infants from Leiden and surrounding communities in the Netherlands visited the laboratory (age *M* = 216 days; SD = 4.9 days; 43.2% girls), and 69 of them returned to the 12‐month assessment (age *M* = 375 days; SD = 12.2 days; 47.8% girls). The families were recruited through letters mailed based on contact information obtained from the city council. All mothers were the biological mothers of the infants (mothers’ mean age = 32.8 years, SD = 3.7 years). A majority of the infants came from families where both parents had the Dutch nationality (88.4%). Using a 5‐point scale to assess the education level of both parents (1: primary school, 2: vocational school, 3: secondary school, 4: post‐secondary applied education, 5: university degree), the mean score of both parents was 4.24 (SD = 0.71), indicating a mostly highly‐educated sample.

For the analyses of ERP and attachment associations, six infants were excluded due to having less than 10 good EEG segments per condition (*n* = 2, see Section 2.4) or technical errors (*n* = 4), resulting in 63 infants included in these analyses. For the analyses of dwell time and attachment associations, 17 infants were excluded due to having less than three good trials per each stimulus condition (*n* = 11), not completing the task after the ERP recording (*n* = 3), or experimenter error (n = 3), leaving 52 infants in the analyses of dwell time and attachment data. Of the 19 infants who dropped out between 7 and 12 months (out of the original *n* = 88), 15 and 10 infants had sufficient ERP and dwell time data, respectively. No significant differences were observed in the ERP amplitudes or dwell times between those who dropped out versus those who remained in the study.

### 2.2 Procedure

**FIGURE 1:**
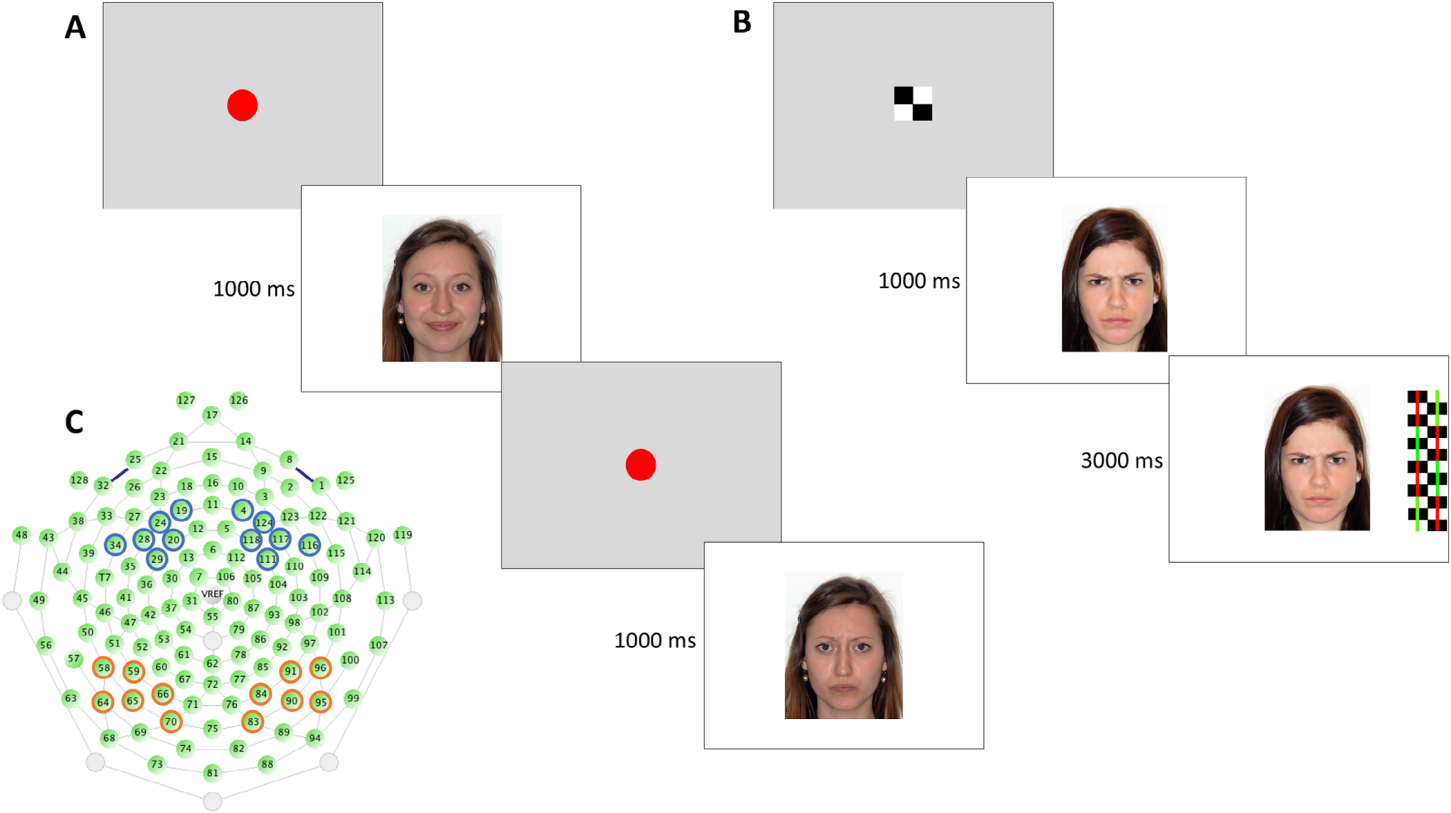
(A) The mother's angry and happy faces presented in random order in the ERP task. (B) Sequence of events on a single trial of the Overlap task. (C) Electrode layout depicting the channel groups for analyzing the N290 and P400 (red circles), and Nc (blue circles) ERP responses.

The 7‐month assessment started with an ERP task measuring brain responses to the mother's face stimuli, followed by an Overlap task measuring infants’ attention dwell times to the mother's and a stranger's facial expressions (Figure 1). Task order was kept constant to maximize the amount of good ERP data. At the end of the visit, observed maternal sensitivity was assessed in a semi‐structured free‐play situation. At 12 months, infant‐mother attachment was assessed with the Strange Situation Procedure (SSP). At both visits mothers signed informed consent, and on the first visit mothers provided signed consent for their pictures to be used in the data collection of the present study. Infants received a small gift and a diploma, and travel costs were reimbursed. The study protocol was approved by the Ethics Committee of the Institute of Education and Child Studies at Leiden University.

### 2.3 Stimuli

The stimuli were obtained by taking pictures of the mother either during a home visit within 2 weeks preceding the laboratory visit or in the beginning of the laboratory visit. A uniform white background was used during photography for all participants. Mothers were asked to remove their earrings and to keep their hair as they usually do. Eyeglasses were permitted. Mothers were shown stereotypical examples of closed mouth angry and happy facial expressions from the NimStim stimulus set (Tottenham et al. 2009) and asked to display a similar expression on their own face as they would naturally do. To select the stranger's face to be used in the Overlap task, an individual most closely matching the mother's face in appearance (i.e., hair color and style, presence of glasses) was selected as the stranger stimuli. To obtain a sufficiently variable pool of stranger faces, pictures were also obtained from 7 individuals from the laboratory. For the laboratory tasks, the pictures were cropped close to the head outline and resized to measure 14° × 11° visual angle when viewed from a 60‐cm distance. Original colors of the stimuli were retained. An independent sample of 27 participants (*M* = 25.4 years) rated the valence of the faces on a 9‐point scale from 1 (highly negative) to 9 (highly positive). Angry faces (*M* = 3.24; SD = 0.73) were rated significantly more negative than happy faces (*M* = 6.72; SD = 0.51), *t*(67) = 31.88, *p* < 0.001, *d* = 0.90, and no difference in the valence ratings was observed between faces of mothers of securely versus insecurely attached infants (angry: *t*(66) = 1.40, *p* = 0.17; happy: *t*(66) = 0.43, *p* = 0.67), or between faces of mothers of infants with organized versus disorganized attachment (angry: *t*(66) = 0.99, *p* = 0.33; happy: *t*(66) = 0.41, *p* = 0.69). In addition, to rule out potential differences in low‐level image properties, stimulus brightness was evaluated by calculating mean RGB values for the stimuli with the SHINE toolbox (Willenbockel et al. 2010). No differences were observed in mean RGB intensity between faces of mothers of securely versus insecurely attached infants (angry: *t*(66) = 1.40, *p* = 0.17; happy: *t*(66) = 0.43, *p* = 0.67), or between faces of mothers of infants with organized versus disorganized attachment (angry: *t*(66) = 0.99, *p* = 0.33; happy: *t*(66) = 0.41, *p* = 0.69).

### 2.4 Measures

#### 2.4.1 ERP Responses

EEG was recorded to measure infants’ ERP responses to the mother's angry and happy facial expressions. We only included face stimuli of the mother due to the risk that including four conditions (angry and happy stimuli from both mother and stranger) would result in excessive attrition, which is a common problem in infant ERP research (Stets et al. 2012). In the ERP task, the infants were sitting in a darkened EEG recording room on their mother's lap approximately 60 cm from a 17‐inch computer monitor that was surrounded by black curtains. They were presented with a maximum of 120 trials (*M* = 105.50; SD = 21.12) displaying their mother's angry or happy faces in random order (i.e., a maximum of 60 trials for each facial expression). The task was discontinued if the infant became fussy and inattentive. Each trial was initiated by the experimenter who monitored the infant from an adjacent room via a video camera mounted above the stimulus presentation monitor, which also recorded the infant's behavior during the task. On each trial, the face stimulus was presented on the center of the screen with a white background for 1000 ms, followed by a dynamic attention getter (a pulsating red circle) which was shown until the experimenter initiated the next trial (Figure 1A). To prevent habituation, after every 6 trials a 4‐s animation was presented, which was randomly selected from five different animations of moving toys and animals.

Infants’ EEG was recorded using a 124‐channel Hydrocel Geodesic Sensor Net, amplified with a NetAmps300 amplifier, low‐pass filtered at 100 Hz, and digitized at a rate of 250 Hz using NetStation software (Electrical Geodesics Inc., Eugene, OR). Impedances were kept below 50 kΩ and the continuous EEG signal was referenced to Cz. Before exporting the EEG recording for offline analyses, each infant's video recording was screened and point markers were added to the EEG recording to mark all segments during which the infant looked away from the screen. The EEG data were then high‐pass filtered at 0.3 Hz and exported.

Offline, the EEG data were processed with Brain Vision Analyzer 2.2 (Brain Products GmbH). The data were first low‐pass filtered at 30 Hz (48 dB/octave) and re‐referenced to the average of all recorded electrodes. In cases with a small number (maximum 10) of faulty or consistently bad electrodes, these electrodes were excluded from the average reference calculation, but the new reference was applied to all electrodes. The EEG was then segmented to 1100‐ms long epochs containing the stimulus presentation and a 100‐ms baseline preceding stimulus onset, and the segments were baseline‐corrected. Next, each segment was visually inspected for artifacts related to eye movements, blinks, and not looking at the screen. Segments marked as bad during video inspection and segments containing clear ocular artifacts on channels around the eyes (1, 8, 14, 21, 25, and 32) were removed. To detect residual artifacts, automatic artifact detection was run on each channel, marking the channels as bad if the amplitude within the segment exceeded ±150 µV, or if the activity was lower than 0.5 µV within any 100‐ms interval. Channels containing artifacts were then removed from individual segments. Infants who provided at least 10 good trials in each stimulus condition (cf. Conte et al. 2020) were included in the analyses. On average the infants included in the analyses provided 27.3 (SD = 10.1) and 27.6 (SD = 10.4) good trials for the happy and angry stimulus conditions, respectively.

ERP components were extracted from occipitotemporal (N290 and P400) and frontocentral (Nc) electrode sites (Figure 1C), with the time windows and included electrodes based on previous studies (e.g., Conte et al. 2020; Peltola et al. 2020) and visual inspection of the grand average graphs for electrodes showing the most prominent ERP waveforms of the components of interest. We focused on amplitude‐based measures in the analyses as they are considered more reliable than latency‐based ERP measures (Huffmeijer et al. 2014; Munsters et al. 2019). The N290 and P400 were quantified as the minimum and maximum amplitudes within time‐windows of 200–350 ms and 300–450 ms, respectively, from electrodes in the left (58, 59, 64, 65, 66, and 70) and right (83, 84, 90, 91, 95, and 96) hemisphere occipitotemporal cortex. The time windows overlapped slightly to ensure that the peak activity during the N290 and P400 waveforms was sufficiently covered for all participants. The Nc was quantified as the mean amplitude between 300 and 550 ms from channels covering the left (19, 20, 24, 28, 29, and 34) and right (4, 111, 116, 117, 118, and 124) hemisphere frontocentral area.

#### 2.4.2 Attention Dwell Time

After removing the electrode net, the infants moved to another room where they were presented with the Overlap task (Peltola et al. 2013; Peltola et al. 2018) to measure infants’ attention to the mother's and a stranger's happy and angry faces. Infants sat on their mother's lap inside a curtained booth at a distance of 60 cm from a 42‐inch monitor, which had a video camera mounted on top to monitor and record the infant's looking behavior. In the Overlap task, a face stimulus (14° × 11°) was first presented on the center of the screen for 1000 ms after which it was flanked by a 14° × 4° distractor stimulus (a black‐and‐white checkerboard or circle pattern overlaid by red and green stripes) on the left or right side of the face for 3000 ms (Figure 1B). The face stimulus on each trial was either the mother's or a stranger's happy or angry face, with 6 repetitions of each stimulus (i.e., a total of 24 trials) that were presented in random order. Each trial was started manually by the experimenter when the infant looked at a checkerboard fixation stimulus on the center of the screen.

Attention dwell times were manually derived from video recordings with frame‐by‐frame playback (Peltola et al. 2013, 2008). Trials with excessive movement during the trial, attention not directed at the screen at the beginning of the trial, anticipatory attention shifts (eye movements commenced within 160 ms after distractor onset), and trials with eye movements away from the face that were not directed toward the distractor stimulus were excluded from the analyses. Infants with less than 3 accepted trials per stimulus condition were excluded from the analyses (Leppänen et al. 2018). Infants included in the analyses had on average 19.93 (SD = 2.62) accepted trials (mother happy = 4.92; mother angry = 5.02; stranger happy = 4.98; stranger angry = 5.02). For statistical analyses, the duration of attention dwell time on the face stimulus was determined for the period starting 160 ms from distractor stimulus onset and ending 1000 ms after distractor stimulus onset. Dwell time duration was then converted to a normalized dwell time index score (Leppänen et al. 2018; Peltola et al. 2018) by using the following formula:

$$\mathrm{Dwell}\;\mathrm{time}\;\mathrm{index}=\frac{\sum_{i=1}^{n}\;\left(1-\frac{1000-x_{i}}{840}\right)}{n},$$

where *x* is the time point at which the eye movement from the face towards the distractor stimulus starts and *n* is the number of scorable trials in a given stimulus condition. In this index, the shortest acceptable saccadic eye movement latency (160 ms) results in a score of 0, and the longest possible latency (or a lack of an attention shift, which is equal to the last measured time point at 1000 ms) in a score of 1. The benefits of the normalization as compared to calculating average saccadic latencies are that this method does not require excluding trials without any gaze shifts, which are common in young infants (Leppänen et al. 2018; Peltola et al. 2015, Peltola et al. 2018), and by using a 1000‐ms cut‐off it reduces the influence of very late gaze shifts on the mean dwell times (Leppänen et al. 2018).

#### 2.4.3 Maternal Sensitivity

At the end of the 7‐month visit, maternal sensitivity was assessed during a semi‐structured 5‐min free play interaction, during which the mother–infant dyad was provided with a box of age‐appropriate toys and instructed to interact as they normally would. The interaction was recorded and maternal sensitivity was coded with the Ainsworth scales for Sensitivity and Cooperation (Ainsworth et al. 1974) with scores ranging from 1 (highly insensitive/highly interfering) to 9 (highly sensitive/highly cooperative). The videos were coded by an expert coder and a trained coder. On a set of 15% of the videos coded by both coders, adequate levels of reliability were observed for sensitivity (ICC, absolute agreement = 0.74) and cooperation (ICC, absolute agreement = 0.66). Sensitivity (*M* = 5.3; SD = 1.88) and Cooperation (*M* = 4.48; SD = 1.89) scores were highly correlated, *r* = 0.80, *p* < 0.001, and were therefore standardized to account for mean level differences and averaged into one score indicating parental sensitivity, in line with previous research (e.g., Bakermans‐Kranenburg et al. 2022).

#### 2.4.4 Attachment

At 12 months, infants’ attachment quality was assessed with the Strange Situation Procedure (SSP; Ainsworth et al. 1978). The SSP consisted of eight episodes, which included two separations from and two reunions with the mother, and interaction with a female stranger (i.e., a previously not seen experimenter). Attachment behavior during the two reunion episodes was assessed from video recordings by two expert coders who were blind to other information about the infants. The organized attachment scales by Ainsworth et al. (1978) and the Main and Solomon (1990) coding system for assessing attachment disorganization were used for coding infants’ attachment behaviors. Using 7‐point scales assessing infants’ proximity seeking, contact maintenance, resistance, and avoidance during the two reunion episodes, infants were first classified as secure (B), insecure‐avoidant (A), or insecure‐resistant (C). Next, behaviors indicating attachment disorganization (D) during each episode when the mother was present were rated using a scale from 1 (no signs of disorganization) to 9 (strong signs of disorganization), and a cut‐off of 5.5 was used to classify for disorganized attachment. The attachment disorganization scoring focuses on contradictory behaviors (e.g., rapid avoidance following a cry for the mother), stereotypical or anomalous behaviors, stilling or freezing, direct signs of apprehension regarding the mother, and misdirected or disoriented behaviors. Twenty randomly selected additional SSPs that were reported in a previous study from the same laboratory (Biro et al. 2017) were coded by both coders. Intercoder agreement for these cases was 75 % (*κ* = 0.62, *p* = 0.001) for the ABC classifications and 85 % (*κ* = 0.69, *p* = 0.002) for disorganized versus organized (ABC) attachment categories. In the sample of 69 infants assessed for attachment at 12 months, 37 infants were classified as securely attached (B), 4 as insecure‐avoidant (A), 15 as insecure‐resistant (C), and 13 as disorganized (D), thus fairly closely resembling meta‐analytic global distributions of SSP classifications (Madigan et al. 2023). In addition, continuous attachment security scores were computed based on the interactive scale scores according to the algorithm of Van IJzendoorn and Kroonenberg (1990). Intra‐class correlation coefficients for the continuous measures were 0.82 for the security score and 0.77 for the disorganization score (Biro et al. 2017).

### 2.5 Statistical Analyses

The data were missing completely at random (Little's MCAR test: *χ* ^2^(163) = 118.258, *p* = 0.997). The ERP data were analyzed using a 2 × 2 × 2 mixed analysis of variance (ANOVA) with Emotion (happy, angry) and Hemisphere (left, right) as within‐subjects factors and Attachment (secure, insecure) as a between‐subjects factor, and maternal sensitivity as a covariate. The attention dwell time data were analyzed with a 2(Familiarity: mother, stranger) × 2(Emotion: happy, angry) × 2(Attachment: secure, insecure) ANOVA, with maternal sensitivity as a covariate. In additional analyses exploring potentially specific associations with attachment disorganization, the same ANOVAs were run but with Attachment Organization (organized, disorganized) as a between‐subjects factor. When observing interactions involving attachment, follow‐up analyses were run separately within the two groups, and also by comparing responses between groups with independent sample *t*‐tests. Finally, for exploratory purposes, bivariate correlations between the continuous attachment scores (security and disorganization scores) and the key variables are reported. These correlations inform about whether continuous measures of attachment were associated with infants’ neural and attentional responses to faces, and maternal sensitivity.

## 3 Results

Descriptive information of the variables included in the analyses is presented in Table 1.

**TABLE 1:** Descriptive statistics of the key variables for the attachment groups (secure vs. insecure).

	Secure		Insecure	
Variable	*n*	*M* (SE)	*n*	*M* (SE)
Maternal sensitivity	37	−0.087 (0.16)	32	0.13 (0.17)
Security score	37	2.0 (0.23)	32	−1.15 (0.41)
Disorganization score	37	2.25 (0.29)	32	3.81 (0.41)
N290 happy left	32	1.68 (1.27)	31	2.62 (1.34)
N290 happy right	32	0.22 (0.96)	31	3.07 (0.97)
N290 angry left	32	0.47 (1.50)	31	2.58 (1.64)
N290 angry right	32	1.61 (0.99)	31	4.36 (1.39)
P400 happy left	32	18.98 (1.94)	31	19.90 (1.64)
P400 happy right	32	17.97 (2.03)	31	20.77 (1.58)
P400 angry left	32	17.57 (2.02)	31	20.15 (1.65)
P400 angry right	32	17.85 (1.97)	31	23.01 (1.75)
Nc happy left	32	−3.68 (1.15)	31	−3.43 (0.83)
Nc happy right	32	−4.28 (0.99)	31	−4.21 (0.78)
Nc angry left	32	−1.56 (0.86)	31	−4.67 (0.96)
Nc angry right	32	−2.59 (0.94)	31	−4.11 (0.79)
DT mother happy	29	0.53 (0.04)	23	0.43 (0.03)
DT mother angry	29	0.52 (0.04)	23	0.44 (0.04)
DT stranger happy	29	0.58 (0.04)	23	0.59 (0.04)
DT stranger angry	29	0.58 (0.04)	23	0.54 (0.05)

*Note*: Maternal sensitivity represents standardized average score of sensitivity and cooperation. ERP amplitudes are reported as microvolts and dwell times (DT) as standardized dwell time index scores ranging from 0 to 1.

### 3.1 ERP Responses

**FIGURE 2:**
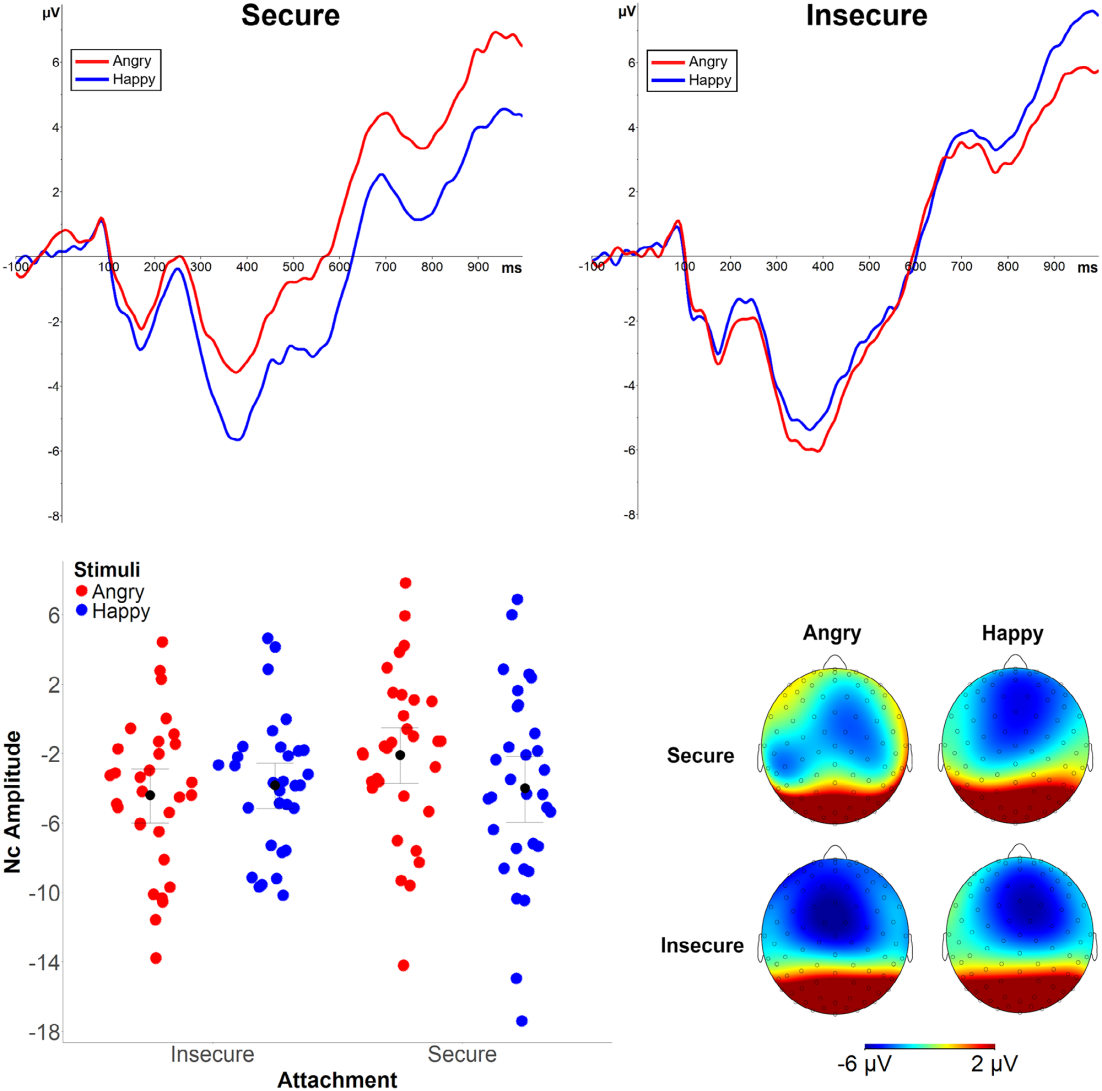
Top panels: Nc ERP waveforms (300–550 ms) to the mother's angry and happy faces in frontocentral electrodes (averaged across the hemispheres) in infants with secure (top left graph) and insecure (top right graph) attachment. Amplitude (µV) is displayed on the y‐axis and time (ms) on the x‐axis. The bottom graphs represent individual data points of Nc amplitudes (bottom left graph) and the scalp topographies (bottom right graph) displaying average activity distribution during the time window of the Nc component (300–550 ms).

In an ANOVA of the Nc mean amplitudes, we observed an Emotion x Attachment interaction, *F*(1, 60) = 4.53, *p* = 0.038, $\eta_{p}^{2}$ = 0.07, but no main effects of Hemisphere, Emotion, Attachment, or Maternal Sensitivity, or other interactions involving Attachment (*F*s < 1.74). To break down the significant Emotion x Attachment interaction, the secure and insecure groups were first analyzed separately. As can be observed from Figure 2 and Table 1, the securely attached infants had larger (i.e., more negative) Nc amplitudes to the mother's happy versus angry faces, *F*(1, 31) = 4.25, *p* = 0.048, $\eta_{p}^{2}$ = 0.12. In insecurely attached infants, no difference between Nc amplitudes to maternal happy versus angry faces was observed, *F*(1, 30) = 0.67, *p* = 0.42, $\eta_{p}^{2}$ = 0.02. In another follow‐up analysis comparing Nc amplitudes to angry and happy faces (averaged across the hemispheres) between groups, the groups did not differ in responses to maternal happy faces, *t*(61) = 0.13, *p* = 0.89, *d* = 0.03, but insecurely attached infants showed significantly more negative Nc amplitudes to maternal angry faces than securely attached infants, *t*(61) = 2.03, *p* = 0.047, *d* = 0.51. In an ANOVA comparing infants with organized versus disorganized attachment, a similar Emotion × Attachment Organization interaction was observed, *F*(1, 60) = 4.17, *p* = 0.046, $\eta_{p}^{2}$ = 0.07. The within‐group differences in Nc amplitudes to angry and happy faces were not significant in either the organized (*M*_angry_ = −2.61 µV; *M*_happy_ = −3.91 µV) (*p* = 0.06) or the disorganized group (*M*_angry_ = −5.52 µV; *M*_happy_ = −3.87 µV) (*p* = 0.15). In the between‐groups comparisons, Nc responses to angry faces were significantly larger (i.e., more negative) in the disorganized group, *t*(61) = 2.07, *p* = 0.043, *d* = 0.64, whereas no group difference was found for happy faces, *t*(61) = 0.02, *p* = 0.98, *d* = 0.01.

**FIGURE 3:**
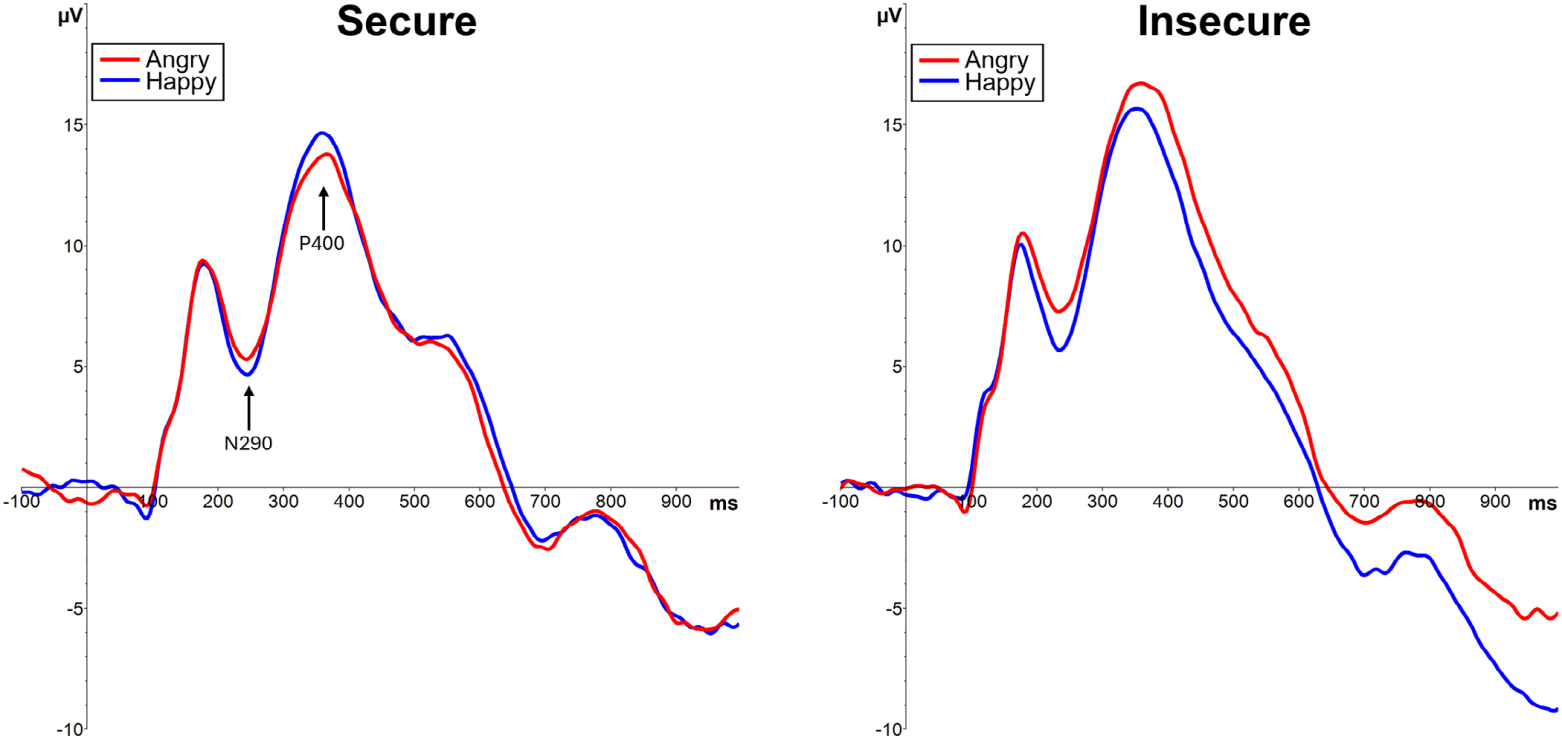
N290 and P400 ERP responses to the mother's angry and happy faces in occipitotemporal electrodes (averaged across the hemispheres) in infants with secure (left graph) and insecure (right graph) attachment. Amplitude (µV) is displayed on the y‐axis and time (ms) on the x‐axis.

Regarding the N290 and P400 peak amplitudes, no significant interactions involving Attachment or Maternal Sensitivity, or main effects were observed for either of the components (*F*s < 3.79; see Table 1 for descriptive data and Figure 3 for the ERP graphs). When comparing infants with organized versus disorganized attachment, only a Hemisphere × Emotion interaction in the N290 amplitudes was significant, *F*(1, 60) = 4.55, *p* = 0.04, $\eta_{p}^{2}$ = 0.07, due to N290 amplitudes being larger (i.e., more negative) to happy faces on the right but not the left hemisphere channels. No significant effects for the P400 and no interactions involving Attachment Organization were found for either of the components (*F*s < 3.21).

### 3.2 Attention Dwell Time

**FIGURE 4:**
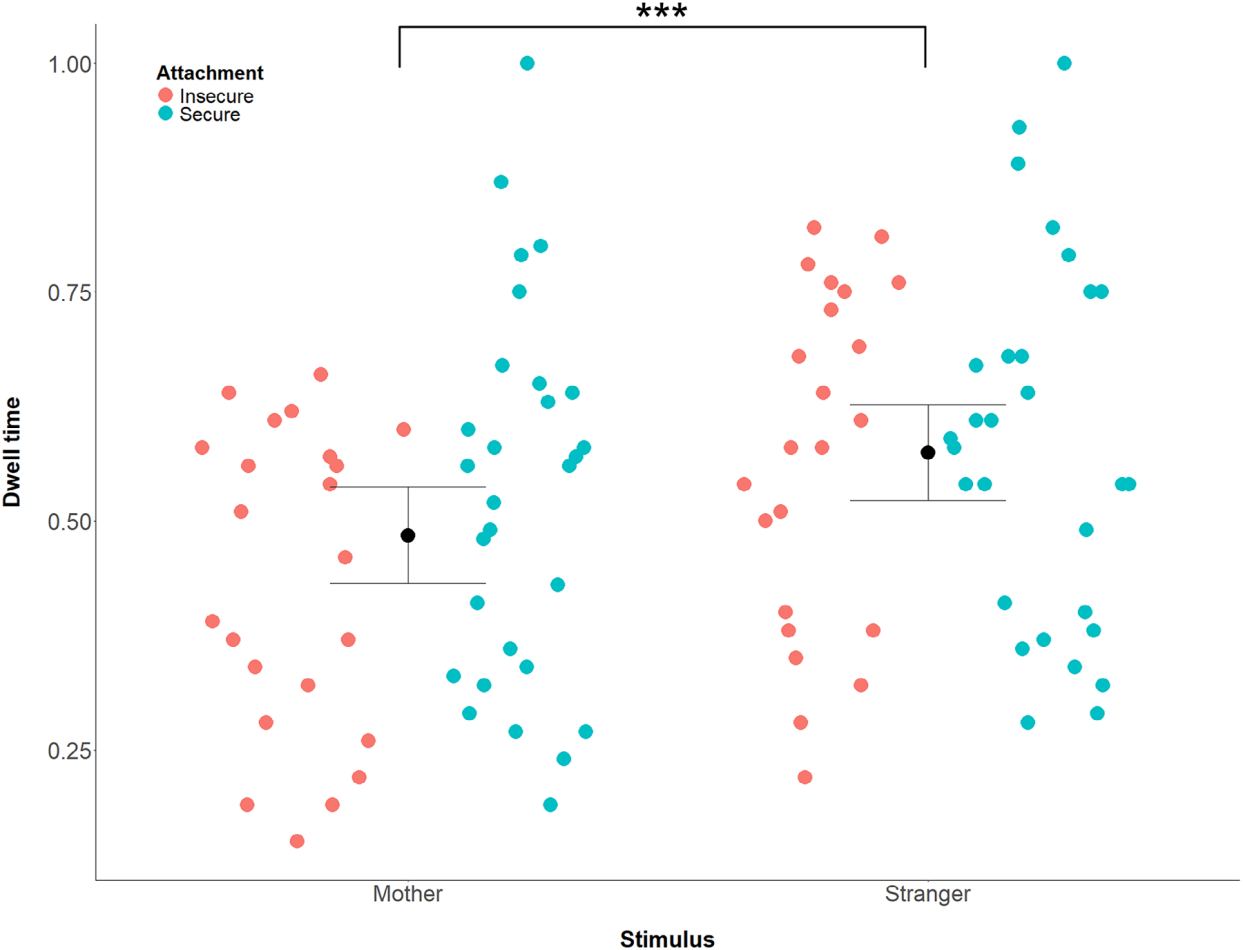
Attention dwell times to mother and stranger faces (averaged across angry and happy faces) in infants with insecure and secure attachment. The asterisks indicate the main effect of familiarity, that is, the difference between dwell times to mother and stranger faces.

An ANOVA of the attention dwell times in the Overlap task produced a significant main effect of Familiarity, *F*(1, 49) = 19.66, *p* < 0.001, $\eta_{p}^{2}$ = 0.29, due to the dwell times being longer to the stranger's (*M* = 0.57) as compared to the mother's (*M* = 0.48) face stimuli across infants. While this difference appeared to be more pronounced in insecurely attached than securely attached infants (Table 1 and Figure 4), the interaction between Familiarity and Attachment was only marginal, *F*(1, 49) = 3.67, *p* = 0.06, $\eta_{p}^{2}$ = 0.07, and thus the follow‐up tests are not reported. The other main effects and interactions regarding Attachment or Maternal Sensitivity were non‐significant (*F*s < 1.79). Similarly, in an ANOVA comparing infants with organized versus disorganized attachment, only the main effect of Familiarity was significant, *F*(1, 49) = 14.06, *p* < 0.001, $\eta_{p}^{2}$ = 0.22.

### 3.3 Associations With Continuous Attachment Scores

The continuous attachment security scores (Van IJzendoorn and Kroonenberg 1990) were not significantly associated with the ERP amplitudes, attention dwell times or maternal sensitivity scores. The disorganization scores were negatively correlated with infants’ Nc amplitudes to angry faces in the left, *r*(63) = −0.25, *p* = 0.049, and right hemisphere channels, *r*(63) = −0.28, *p* = 0.026, indicating that greater disorganization scores were related to larger Nc responses to the mother's angry expression. Disorganization scores were not associated with other ERP amplitudes, attention dwell times or maternal sensitivity scores.

## 4 Discussion

This longitudinal study extended research on attachment‐related processing of social and emotional information in infancy by investigating whether 7‐month‐old infants’ neural and attentional responses to their mother's angry and happy facial expressions are associated with infants’ attachment security to the mother at 12 months of age. Neural responses to maternal angry and happy faces were measured with an ERP task and attention to maternal and stranger emotion expressions was assessed with an Overlap task, which measured infants’ dwell times to faces in the presence of peripheral distractor stimuli. Maternal sensitivity during free play was assessed at 7 months and included as a covariate in the main analyses. Infants’ attachment was assessed at 12 months with the Strange Situation procedure.

The key results indicated that infants’ attention‐related brain responses to maternal facial expressions are associated with attachment security and disorganization. In the main analysis comparing securely versus insecurely attached infants, amplitudes of the Nc ERP component were larger to maternal happy than angry expressions in infants who were later classified as having secure attachment, whereas no difference in Nc amplitudes to maternal facial expressions was observed in infants with later insecure attachment. Importantly, the rating data and analysis of low‐level image properties indicated that the differences in ERP responses were not due to differences in the intensity or low‐level properties of the facial expression stimuli between different attachment groups. The current result is highly similar to that of Kungl et al. (2023) who found that in school‐aged children, attention‐related ERP responses (i.e., LPP component amplitudes) to maternal happy versus angry facial expressions did not differ in children with insecure attachment, whereas children with secure attachment showed larger ERP amplitudes to maternal happy versus angry faces. Considering these results together, it is possible that already before the first birthday, children who are later classified as securely attached to their caregiver are particularly sensitive to signals of affiliation and positive affect expressed by their attachment figure, which translates to more pronounced attention‐related neural responses to such signals than to signals of rejection and negative affect by the attachment figure. This may indicate greater approach motivation triggered by the reward value of happy faces in securely attached infants through repeated experiences of positive interaction with the caregiver (Kungl et al. 2023; Long et al. 2020). Such an acquired information processing bias would also be predicted by the recently introduced Learning Theory of Attachment by Bosmans et al. (2020), which would also predict that such sensitivity to rewarding social signals extends outside the cues expressed in a specific attachment relationship. As our ERP measurement only included own mother's faces, we cannot ascertain whether similar ERP modulation by happy faces in infants with secure attachment would be observed with unfamiliar faces.

In another follow‐up test of the interaction between attachment and emotion in the Nc ERP data, the secure and insecure attachment groups differed in Nc amplitudes to angry faces, but not happy faces, indicating that infants with insecure attachment showed stronger, and secure infants relatively reduced, attention‐related neural responses to maternal angry faces. Moreover, in the correlation analyses, greater attachment disorganization scores were correlated with larger Nc responses to maternal angry faces. As each infant saw only faces of their own mother in the current ERP measurements, we consider the between‐groups comparisons secondary to the within‐groups analyses in terms of evidentiary value. Nevertheless, the findings may indicate that infants with insecure attachment and particularly those with increased levels of attachment disorganization are more vigilant to cues of anger in maternal facial expressions. Larger cortical responses to angry faces in infants with insecure attachment and higher disorganization could be seen to be partially at odds with the results of Peltola et al. (2015), in which infants with insecure and disorganized attachment showed a smaller attention bias to unfamiliar fearful faces in the Overlap task as compared to infants with secure and organized attachment. Although direct comparison between these two studies should be made cautiously because of differences in stimulus familiarity and the attention measure (ERP responses vs. attention disengagement), it is possible that emerging patterns of attachment security and organization during early development are associated with social information processing in emotion‐specific ways. Clearly, as the number of studies is still very limited, more research is needed to uncover whether specific emotional signals of the attachment figure show replicable response patterns in infants as a function of attachment security and organization.

In the current data, no associations with attachment were observed in the N290 and P400 ERP responses. Particularly the N290 relates to perceptual discrimination of faces and originates from face‐sensitive structures such as the fusiform gyri (Conte et al. 2020; Yrttiaho et al. 2022). In this respect the results differ from those of Peltola et al. (2020) who observed that the N290 responses discriminated between fearful and non‐fearful faces in securely attached infants, but not in infants with insecure or disorganized attachment. Also in this case we hesitate to draw strong conclusions regarding the seemingly discrepant results given the differences in emotional content and familiarity of the stimuli, as well as experimental paradigms, between the studies. It should also be noted that, as reviewed in the introduction, the overall pattern of emotional modulation of the N290 and P400 responses is not clear, with mixed results regarding how fearful versus angry facial expressions impact these components (Kobiella et al. 2008; Xie et al. 2019). It remains to be further explored whether the impact of variations in attachment security on social information processing in early childhood is observed in relation to both attentional and perceptual processes.

Results regarding attention dwell times in the Overlap task were inconclusive. The absence of a main effect of emotion replicates the findings of Leppänen et al. (2018) who observed no differences in dwell times between happy and angry faces in the Overlap task at 5, 7, and 12 months of age, while a robust difference between happy and fearful faces was found. The key outcome in the current data was a robust influence of familiarity on attention, with the dwell times being longer to the stranger faces. This likely reflects the novelty of the stranger faces capturing infants’ attention, particularly as the task included the same maternal face stimuli that were used in the ERP task preceding the Overlap task. While the mean attention dwell times suggested that greater attention to the stranger faces might have been driven by reduced attention to the mother faces in infants with insecure attachment (Figure 4), the interaction between attachment and stimulus familiarity was marginal, and therefore this pattern could not be verified. Nevertheless, such pattern would be consistent with findings in older children that have shown longer looking at the mother's than unfamiliar women's face stimuli in securely attached school‐age children (Vandevivere et al. 2014). The absence of a clear interaction may also be due to the very large main effect of stimulus familiarity, thus leaving little room for variation related to attachment. Further investigation of attachment‐related variation in infants’ processing of emotion signals displayed by the actual attachment figures is important, as it provides opportunities to test whether attachment‐related differences in processing social signals of unfamiliar people (e.g., Peltola et al. 2015) are preceded by differentiated responses to social signals of the caregivers. This would be in line with studies investigating the impact of face familiarity on the discrimination of facial expressions in infancy, which indicate that infants first learn to discriminate expressions displayed by caregivers before such skills are generalized to facial expressions of other people (Montague and Walker‐Andrews 2002; Walker‐Andrews et al. 2011).

In this study, maternal sensitivity did not have an impact on the associations between face processing and attachment, and it was not correlated with the continuous measures of attachment. Despite an established association between parental sensitivity and infant attachment security, the size of the association tends to be modest and vary substantially across studies (De Wolff and van IJzendoorn 1997; Verhage et al. 2016). It has also been proposed that parental sensitivity assessed during infant distress may be more strongly associated with attachment than sensitivity assessed during free play interactions (Leerkes 2011). Regarding associations between maternal sensitivity and face processing, there is evidence pointing to positive associations between maternal sensitivity and Nc ERP responses to happy faces (Taylor‐Colls and Fearon 2015) and greater prefrontal cortex activation to happy faces in infants with more sensitive mothers (Stern et al. 2024). Although similar associations were not evident in the current data, which had potentially limited variation due to the brief assessment of maternal sensitivity, our key finding indicated an association between secure attachment and attention‐related brain responses to maternal happy faces. This encourages investigating further whether early interactive patterns contributing to secure attachment become particularly reflected in how infants respond to positive emotion signals of the caregivers and other people. In addition, future studies may benefit from investigating whether more focal measures of parenting, such as observed mind‐mindedness, relate to infants’ processing of emotional information (e.g., Zeegers et al. 2018).

In conclusion, the results of the current longitudinal study suggest that patterns of attachment are associated with differences in neural processing of emotion signals expressed by the attachment figure, with securely attached infants showing larger attention‐related ERP responses to maternal happy than angry faces, while infants with insecure attachment showed no differences in ERP responses between angry and happy faces. Key limitations of the study concern the ERP measurement design and statistical power. As we only presented faces of the own mother to each infant, we are unable to verify whether the observed effects in the Nc ERP responses are specifically related to processing emotional signals of each infant's own mother or whether similar effects would be observed in response to unfamiliar emotion expressions. Regarding statistical power, the current sample size precluded reliable analyses of specific patterns of attachment (e.g., avoidant vs. resistant), which is an important direction for future studies. Finally, although the number of trials included in the current ERP calculations was larger than what is typically required in infant studies measuring ERP responses to visual stimuli (Stets et al. 2012), another limitation of our study is that no quantitative data quality metrics were calculated for the ERP data. To get a better understanding of the normative range of infant ERP data quality across studies, reporting of more direct data quality metrics such as the standardized measurement error (Luck et al. 2021) is recommended.

Despite these limitations and some results being inconclusive, the current results align with an emerging line of research (Peltola et al. 2015, 2020) demonstrating that attachment‐related variation in processing both familiar and unfamiliar emotional signals emerges early in development, and may be detected with longitudinal designs before the formal assessment of attachment can be conducted from 10 to 12 months onwards. The results are also supportive of a broader developmental literature that has demonstrated that individual differences in processing information from other people's faces in infancy can meaningfully predict later social‐emotional developmental outcomes (Eskola et al. 2023; Grossmann et al. 2018; Peltola et al. 2018), highlighting the central role of the face in transmitting social signals in human interactions across the lifespan.

## Ethics Statement

The study protocol was approved by the Ethics committee of the Institute of Education and Child Studies at Leiden University.

## Conflicts of Interest

The authors declare no conflicts of interest.

## Data Availability

The anonymized data are available upon request from the authors.
